# Tm1: A *Mutator/Foldback* Transposable Element Family in Root-Knot Nematodes

**DOI:** 10.1371/journal.pone.0024534

**Published:** 2011-09-08

**Authors:** Stephen M. Gross, Valerie M. Williamson

**Affiliations:** Department of Nematology, University of California Davis, Davis, California, United States of America; University of Poitiers, France

## Abstract

Three closely related parthenogenetic species of root-knot nematodes, collectively termed the *Meloidogyne incognita*-group, are economically significant pathogens of diverse crop species. Remarkably, these asexual root-knot nematodes are capable of acquiring heritable changes in virulence even though they lack sexual reproduction and meiotic recombination. Characterization of a near isogenic pair of *M. javanica* strains differing in response to tomato with the nematode resistance gene *Mi-1* showed that the virulent strain carried a deletion spanning a gene called *Cg-1*. Herein, we present evidence that the *Cg-1* gene lies within a member of a novel transposable element family (Tm1; Transposon in *Meloidogyne*-1). This element family is defined by composite terminal inverted repeats of variable lengths similar to those of *Foldback* (*FB*) transposable elements and by 9 bp target site duplications. In *M. incognita*, Tm1 elements can be classified into three general groups: 1) histone-hairpin motif elements; 2) MITE-like elements; 3) elements encoding a putative transposase. The predicted transposase shows highest similarity to gene products encoded by aphids and mosquitoes and resembles those of the *Phantom* subclass of the *Mutator* transposon superfamily. Interestingly, the meiotic, sexually-reproducing root-knot nematode species *M. hapla* has Tm1 elements with similar inverted repeat termini, but lacks elements with histone hairpin motifs and contains no elements encoding an intact transposase. These Tm1 elements may have impacts on root-knot nematode genomes and contribute to genetic diversity of the asexual species.

## Introduction

Root-knot nematodes (RKNs), comprising the genus *Meloidogyne*, are obligate parasites causing damage to plant roots [1] and weakening plants by acting as carbon sinks [2], [3]. Worldwide, RKNs represent a serious economic problem, causing a 5–12% reduction in crop productivity [4]. Species within the genus *Meloidogyne* differ widely in their modes of reproduction. The three species generally regarded as causing the most damage globally are part of what has been called the *M. incognita* (*Mi*)-group: *M. incognita*, *M. javanica*, and *M. arenaria*. All three are parthenogenetic species in which the eggs are products of mitosis and develop without fertilization [5], [6], [7]. These asexual species are closely related and are thought to have originated from interspecific hybridization followed by the loss of meiosis [8], [9], [10]. Despite their lack of meiosis, this group of RKNs is evolutionarily successful, capable of parasitizing thousands of plant species and acquiring heritable virulent phenotypes [7]. The mechanisms by which these asexual RKN species acquire the ability to parasitize additional host taxa are unknown, but genomic change mediated by transposable elements and repetitive sequences has been proposed [11], [12].

Transposable elements are potent mutagens and can shape genomes in many ways, ranging from large-scale chromosomal rearrangements to subtle alterations in gene regulation [13], [14]. Two general mechanistic classes of transposable elements exist [15]: Class I—retrotransposons, which replicate and insert in new genomic locations through an RNA intermediate; and Class II—DNA transposons, which transpose through cut-and-paste mechanisms without an RNA intermediate. Typical autonomous Class II elements encode a transposase gene flanked by terminal inverted repeats (TIRs). The transposase protein recognizes the TIRs and facilitates mobilization of the element to a new genomic location. Non-autonomous elements do not encode a functional transposase, but can be mobilized in *trans* by transposase proteins produced by an autonomous element. Most transposable elements are also flanked by target site duplications (TSDs) produced as a byproduct of the transposon integration process [16].

A widely studied example of the genetic variability within the apomictic RKNs is the ability to acquire virulence by escaping recognition by tomato plants with the root-knot nematode resistance gene, *Mi-1. Mi-1* confers resistance that is broadly effective against the related *Mi*-group asexual root-knot nematode species [17], [18] and, surprisingly, some isolates of potato aphid and whitefly [19], [20]. However, *Mi-1* does not confer resistance to the RKN species *M. hapla*, a facultative parthenogen in which gametes are products of meiosis, but in the absence of males, sister nuclei re-fuse to produce parthenogenetic diploid progeny [21], [22]. Populations of *Mi-1-*resistance breaking asexual nematodes have been found both in the field and after greenhouse selection [23], [24], [25], [26], [27], [28]. Gleason *et al.* [24] sought to identify genetic differences between an avirulent isolate of *M. javanica* (VW4) and a greenhouse-derived virulent derivative, VW5. Results of both gDNA and cDNA AFLP analyses were consistent with these two nematode strains being isogenic with the exception of the deletion spanning a putative gene named *Cg-1* in VW5. The known 727 bp of the *Cg-1* locus was found to express 456 nt-long mature transcript without obvious coding potential, capable of producing peptides of only 3–32 amino acids, and containing a histone-hairpin motif near its 3′ end. Except for its discovery in *Cg-1*, this motif has been identified only in the 3′ UTR of metazoan, but not plant, histone genes where it is required for processing of histone mRNAs and export of these transcripts from the nucleus [29], [30]. Southern analyses and PCR demonstrated that *Cg-1* is a member of a gene family with 8 copies in the *M. javanica* genome, and transcript analysis indicated that several of these sequences are expressed. Soaking preinfective juveniles of VW4 in dsRNA corresponding to parts of the *Cg-1* sequence resulted in a virulent phenotype capable of parasitizing *Mi-1* tomato, supporting a role for *Cg-1* in *Mi-1*-mediated recognition by the resistant host. However, because of the apparent lack of coding potential, the molecular function of *Cg-1* and its role in mediating response to tomato with *Mi-1* has remained elusive.

Recently, the genome sequences of two root-knot nematodes species, *M. incognita* and *M. hapla*, became available and provide a resource for further investigation of the *Cg-1* locus [10], [31]. Herein we present a detailed analysis of the *Cg-1* locus, and demonstrate that the previously described *Cg-1* gene is within a novel class II transposable element with structurally complex terminal-inverted repeats similar to those of *Foldback* (*FB*) transposable elements. We have named this element Tm1 (for Transposon in *Meloidogyne-*1). We analyze and contrast the Tm1 transposon families of *M. incognita* and *M. hapla*. While Tm1 elements display evidence of transposition activity during the radiation of RKN species, our analyses suggest Tm1 elements are currently inactive and a decaying transposon family in *M. incognita*, *M. javanica*, and *M. hapla*. We propose that despite the lack of obvious recent transposition activity, Tm1 elements may be part of the molecular toolkit asexual root-knot nematodes use to acquire genetic diversity.

## Results

### The *Cg-1* locus contains a transposon-like structure

**Figure 1:**
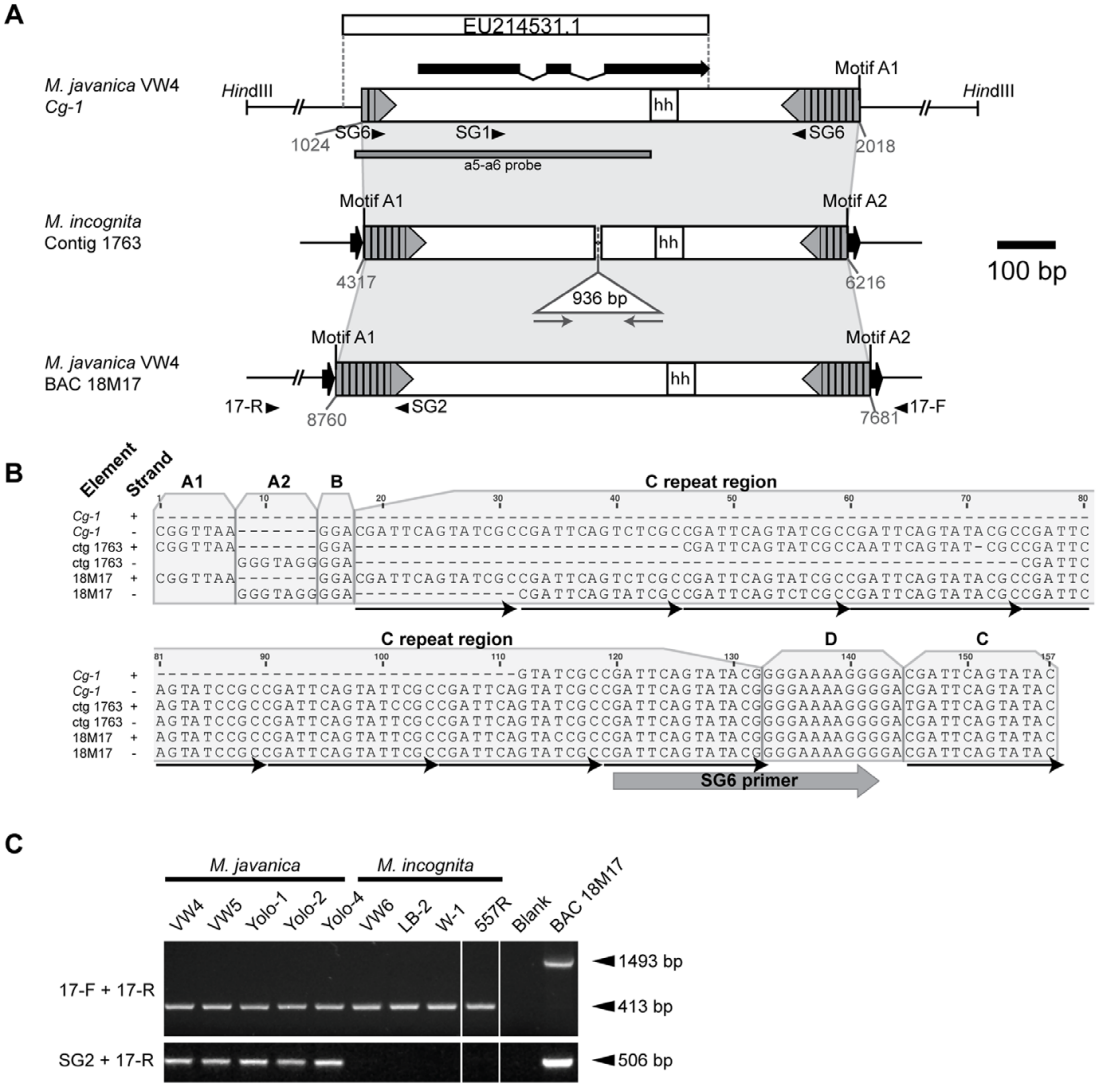
Inverted-repeat element carrying *Cg-1* and related elements. (A) Alignment between elements (open boxes) at the *M. javanica* VW4 *Cg-1* locus (GenBank EU214531.2), *M. incognita* contig 1763 (CABB01001763.1), and *M. javanica* VW4 BAC 18M17 (HQ122410.1). Similar regions between elements are designated with light grey shading. Position of *Cg-1* transcript [24] is shown with thick black lines representing exons and thin lines, introns. Dark grey arrows within open boxes represent the TIRs with vertical bars indicating internal repeats. Target site duplications are indicated black arrows; hh designates the position of the histone hairpin. Binding sites for primers SG2, SG6, 17-F and 17-R are indicated by arrowheads. The grey bar (a5–a6 probe) shows the position of the PCR amplicon used for Southern analysis. (B) Alignment of TIRs of the elements shown in (A). Strand designations+and−represent the 5′ and 3′ ends for the elements as drawn above. Motifs and binding site of primer SG6 are indicated. Thin black arrows below the alignment denote repeat units of Motif C. (C) Agarose gel showing amplification products from genomic DNA of *M. incognita* and *M. javanica* isolates using primers 17-F and 17-R for amplification of empty sites and primer set SG2 and 17-R to detect an insertion similar to that on BAC 18M17.

We used the available 727 bp of the *Meloidogyne javanica* VW4 *Cg-1* locus (GenBank EU214531.1) [24] to query the published genome sequence of *M. incognita*, a RKN species closely related to *M. javanica*. The highest-scoring BLASTN match (E = 6.70e^−48^) was on *M. incognita* contig 1763 (GenBank CABB01001763.1). We noted that sequences 5′ of the *M. javanica Cg-1* transcript are present on contig 1763 in an inverted-repeat orientation flanked by 9 bp direct repeats, suggestive of a transposable element (Figure 1A). The sequences between these inverted repeats are similar to *Cg-1* with the exception of the presence of a second, unrelated 936 bp inverted-repeat element flanked by 7 bp target site duplications being inserted within the region of homology (Figure 1A). In other words, the sequence on *M. incognita* contig 1763 appears to contain two nested transposable elements: a 936 bp element residing within another novel transposable element sharing sequence identity to *M. javanica Cg-1* (Figure 1A). We designed two PCR primers to test whether *M. javanica Cg-1* was also flanked by inverted repeats. The 3′ end of primer SG1 binds a 5 bp sequence (CAATGA) present in *Cg-1* but absent in other members of the gene family [24]. A second primer, SG6, binds within the presumptive inverted repeat. Using SG1 and SG6, we amplified a 629 bp fragment from genomic DNA of *M. javanica* strain VW4, but not VW5 (data not shown), confirming specific amplification of the *Cg-1* locus and supporting the presence of inverted repeats flanking sequences coding for the *Cg-1* transcript.

To investigate the *M. javanica Cg-1* locus further, we screened a genomic DNA library containing ∼3 kb VW4 *Hin*dIII fragments, a size previously demonstrated to harbor *Cg-1* [24], using primers SG1 and SG6. We obtained a 3154 bp clone containing *Cg-1* (GenBank EU214531.2; Figure 1A), and sequence analysis confirmed that the *Cg-1* sequence is flanked by inverted repeats. Sequences outside the inverted repeat element of *M. javanica Cg-1* diverge completely from those outside the element on *M. incognita* contig 1763, further suggesting that these inverted-repeat sequences correspond to the boundaries of transposable elements. However, the inverted repeats flanking *M. javanica Cg-1* do not have target site duplications and the 5′ TIR appears to be truncated (Figure 1A). The element at *Cg-1* is 82.7% identical to that on *M. incognita* contig 1763 excluding the nested 936-bp insertion.

In an attempt to identify additional sequence flanking *Cg-1*, we screened a BAC library of *M. javanica* VW4 by PCR using primers SG1 and SG6. Despite several attempts, we were unable to identify a clone corresponding exactly to *Cg-1* in the BAC library. However, by lowering the annealing temperature of our PCR reaction (Materials and Methods), we obtained a 26 kb BAC clone (clone 18M17, GenBank HQ122410.1). The sequence of this clone identified an element similar to that found at *Cg-1* (Figure 1A). This element was 1080 bp with terminal inverted repeats flanked by 9 bp target site duplications. The sequence is 84.9% identical to *Cg-1*, but sequences outside the element on BAC 18M17 are different from those found flanking elements at *Cg-1* and *Mi* contig 1763, again suggestive of a transposable element.

The three putative transposable elements shown in Figure 1 are similar in sequence and display no obvious protein-coding potential. Between their inverted repeats, each contains a histone hairpin motif (Figure 1A) [24], [29]. Analysis of the TIRs of each element reveals a composite structure (Figure 1B). The TIRs of the elements on contig 1763 and BAC 18M17 have asymmetric 7 bp motifs at their outer ends, internal to the 9 bp target site duplications, here called Motif A1 (5′-CGGTTAA-3′) and Motif A2 (5′-CCTACCC-3′). Internal to Motif A1 and A2 is a 3 bp sequence, Motif B (5′-GGA-3′). The largest portion of the TIR is composed of a variable number of 13 to 15 bp (most often 14 bp) tandem repeats with the consensus 5′- CGATTCAGTATCCGC-3′ (Motif C). Due to differing numbers of repeats of Motif C, the TIRs at each end of the same element, as well as between elements, differ in size. The internal ends of the TIRs (closer to the element center) contain a conserved 12 bp purine-rich sequence stretch (Motif D; 5′-GGGAAAAGGGGA-3′), followed by one additional unit of Motif C.

To determine whether the nematode genomes carried paralogous loci lacking an element and the associated target site duplication (e.g., empty sites) corresponding to the insertion site of the element on BAC 18M17, we surveyed several *M. javanica* and *M. incognita* isolates by amplifying genomic DNA with primers 17-F and 17-R, which flank the putative transposable element (Figure 1A). We obtained ∼0.4 kb amplicons from all tested RKN isolates (Figure 1C), approximately 1 kb shorter than the 1493 bp amplicon produced from BAC clone 18M17 and corresponding to the expected length for an empty site. Sequencing of the 0.4-kb amplicon from VW4 (GenBank HQ122411.2) confirmed that these fragments correspond to a paralogous locus without the inverted repeat element and target site duplications. A second PCR test using a primer within the inverted-repeat element (SG2) and the flanking 17-R primer (Figure 1A) demonstrates that the tested *M. javanica* isolates, but not the *M. incognita* isolates, contain a paralog with an inverted repeat element similar to that on BAC 18M17 (Figure 1C).

Taken together, the identification of 3 similar elements in distinct genomic contexts, two of which are flanked target site duplications, and the identification of paralogous loci with and without an element support the hypothesis these sequences are transposable elements. We have named this transposon family, members of which have novel TIRs with internally redundant motifs, Tm1 (Transposon in *Meloidogyne*-1). Elements closely resembling those found at *Cg-1*, *Mi* contig 1763, and on BAC 18M17 and containing a histone-hairpin motif will be referred to as Tm1-HH.

### Identification of a Tm1 element encoding a 496 amino acid putative transposase

**Figure 2:**
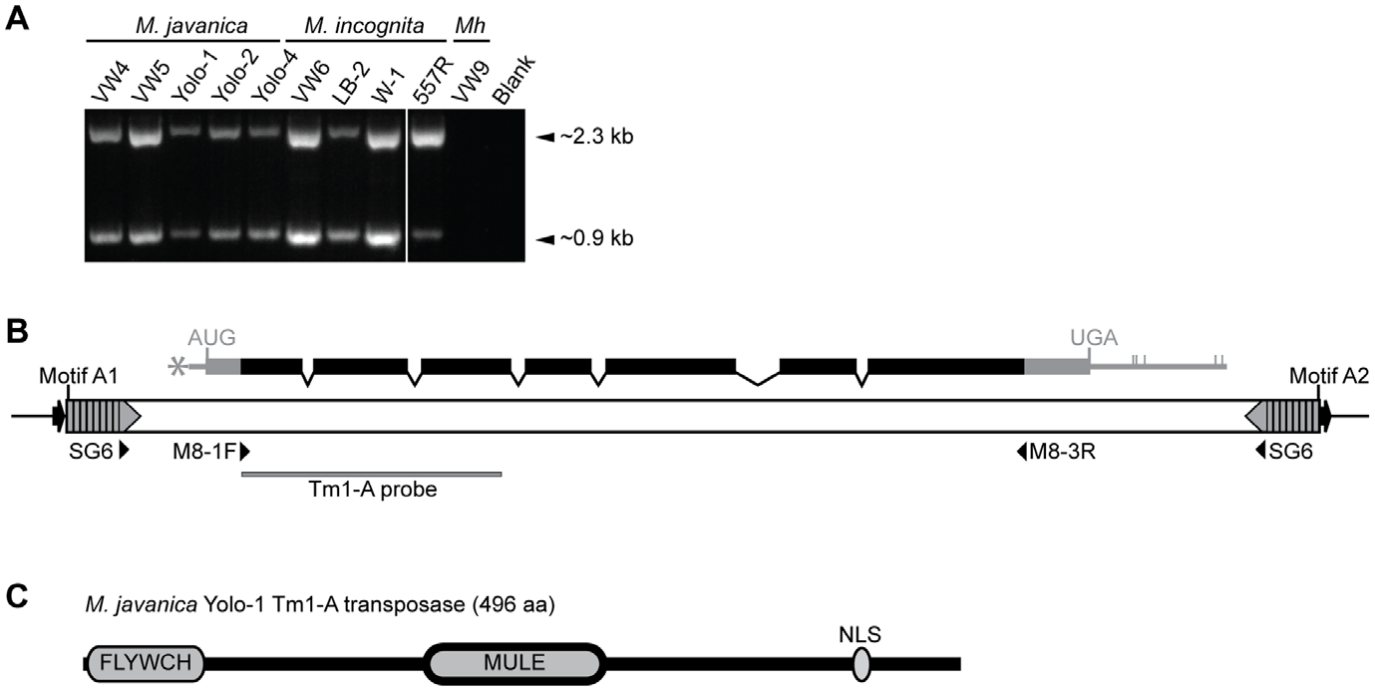
Discovery and analysis of Tm1-A elements. (A) Agarose gel of PCR amplification products with primer SG6 showing the presence of 0.9 kb and 2.3 kb products with *M. javanica* and *M. incognita* isolates. (B) Diagram of 2.5 kb Tm1-A element from *M. incognita* contig 274 (GenBank CABB01000274.1). Transcript, diagrammed above element, is predicted using experimental data acquired from *M. javanica* Yolo-1 (black regions) and genomic sequence (grey regions). Asterisk indicates the predicted 5′ SL1 splice site, and vertical marks, possible polyadenylation sites. All other annotations are as described in Figure 1. “Tm1-A probe” indicates the position of the subclone used for Southern analysis in Figure 3. (C) Predicted 496 aa protein domain protein encoded by the *M. javanica* Yolo-1 Tm1-A element with positions of FLYWCH-MULE domains and nuclear localization signal (NLS) identified by Pfam and PSORT II, respectively, are indicated.

The three Tm1-HH elements discussed previously lack potential to code for a transposase protein and are thus non-autonomous elements. Reasoning that an autonomous Tm1 element is likely to share the same terminal inverted repeats, we amplified nematode DNA using only the SG6 primer, which binds within the TIRs (Figure 1B). We obtained predominant amplicons of approximately 0.9 kb and 2.3 kb in all tested *M. javanica* and *M. incognita* isolates, but no amplicons from the more distantly related *M. hapla* VW9 (Figure 2A). The 0.9 kb amplicons are the size expected from Tm1-HH elements such as those found at *Cg-1* and on BAC 18M17 (Figure 1A). Three of the 2.3-kb amplicons (one each from *M. javanica* VW4, *M. javanica* Yolo-1, and *M. incognita* LB-2) were cloned and sequenced (GenBank accessions HM470231.1, HM470232.1, and HM470230.1, respectively). These three clones were very similar in sequence, with the two *M. javanica* amplicons sharing 99% identity and the *M. javanica* VW4 and *M. incognita* LB-2 amplicons sharing 97% identity. Because the binding site of SG6 was within the TIRs (Figure 1B), the cloned amplicons from VW4, Yolo-1, and LB-2 lack the full TIR. Therefore, we used BLASTN to query the *M. incognita* genome and identified a highly similar element on contig 274 (GenBank CABB01000274.1) (E value = 0). This element is 2593 bp long, and is flanked by 9 bp target site duplications (Figure 2B). The TIRs are structurally similar to those of the Tm1 elements described above and carry the same set of motifs (Figure 2B and Table S1).

A query of the Pfam protein database [32] with the DNA sequences of the 2.5 kb Tm1 element on contig 274 element revealed coding potential for a MULE domain transposase [33], [34]. Thus, we refer to the 2.5 kb Tm1 element as Tm1-A (Tm1-Autonomous). However, no long contiguous ORF was found suggesting the presence of introns. To identify transcripts encoded by Tm1-A elements, we used primers within the predicted coding region (M8-1F and M8-3R; Figure 2B) to amplify partial Tm1-A transcripts from cDNA of *M. javanica* Yolo-1 (GenBank HQ122409.1; Figure 2B) and *M. incognita* LB-2 (not shown). We were not successful in obtaining full-length cDNA, so the 5′ and 3′ ends of the transcript were deduced by comparing the Yolo-1 Tm1-A cDNA sequence to the genomic Yolo-1 Tm1-A sequence. The nearest in-frame start codon (AUG) and stop codon (UGA) flanking the cloned cDNA were found; a spliced leader 1 (SL1) acceptor site (UUUUCAG) [35] was found 5′ of the predicted start codon, and several potential polyadenylation signal sequences (composed of AUUAAA, AAUAAU, GAUAAA, and two repeats of AAAAAA) [35] were identified 3′ of the stop codon. Taken together, the Yolo-1 Tm1-A element is predicted to express a transcript comprised of 7 exons (Figure 2B) and encoding a protein of 496 amino acids (Figure 2C).

The highest BLASTP hits of the predicted full-length 496 amino acid protein encoded by the *M. javanica* Yolo-1 Tm1-A element (GenBank ADM16638.1) correspond to predicted proteins deduced from the genome sequences of aphids and mosquitoes (Table 1). Pfam v. 24.0 [32] revealed that these predicted proteins have a domain structure similar to transposases encoded by *Phantom* elements, a recently described subclass of *Mutator* Class II transposable elements that is widely distributed in eukaryotic taxa including many animals [36] (Figure 2C). Residues 5–66 of the putative Yolo-1 Tm1-A transposase have similarity (E = 1.3e^−7^) to a FLYWCH zinc-finger domain (Pfam PF04500), a protein interaction domain in some isoforms of *Drosophila* Mod(mdg4) proteins [37]. Pfam also identified Yolo-1 Tm1-A residues 197–294 as a MULE domain (E = 9e^−13^) (Pfam PF10551) [33], [34]. MULE domains are found in transposases of the *Mutator* family originally identified in plants [38] and the prokaryotic IS256 family [39].

**Table 1:** Top BLASTP hits of Tm1-A transposase in GenBank.^a^

Accession #	Organism	E Val.	Size (aa)	Description
XP_003243189.1	*Acyrthosiphon pisum*	2e-123	468	hypothetical protein with FLYWCH and MULE domains
XP_003240826.1	*Acyrthosiphon pisum*	3e-87	482	hypothetical protein with MULE domain
XP_003244058.1	*Acyrthosiphon pisum*	1e-85	409	hypothetical protein with MULE domain
XP_003244156.1	*Acyrthosiphon pisum*	3e-40	481	hypothetical protein with FLYWCH and MULE domains
XP_001657863.1	*Aedes aegypti*	1e-36	488	hypothetical protein with FLYWCH and MULE domains
XP_003240985.1	*Acyrthosiphon pisum*	5e-36	483	hypothetical protein with MULE domain
XP_001659669.1	*Aedes aegypti*	1e-35	488	hypothetical protein with FLYWCH and MULE domains
XP_003241855.1	*Acyrthosiphon pisum*	4e-32	433	hypothetical protein with MULE domain
XP_001866309.1	*Culex quinquefasciatus*	6e-32	485	hypothetical protein with MULE domain
XP_001848871.1	*Culex quinquefasciatus*	2e-31	487	hypothetical protein with MULE domain; recombinase-like
YP_001029420.1	*Glypta fumiferanae* ichnovirus	3e-31	404	GfV-C17-ORF1; hypothetical protein with MULE domain; recombinase-like

a Analysis done 08 August 2011.

PSORT II [40] identified two putative overlapping nuclear localization signals (NLSs) near the carboxy end of the Yolo-1 Tm1-A transposase: PPQKARK (residues 438–444) and PQKARKY (439–445). Both are pattern-7 SV40-type NLSs [40], [41] (Figure 2C).

### Tm1 elements are low copy and do not display evidence of recent transposition

**Figure 3:**
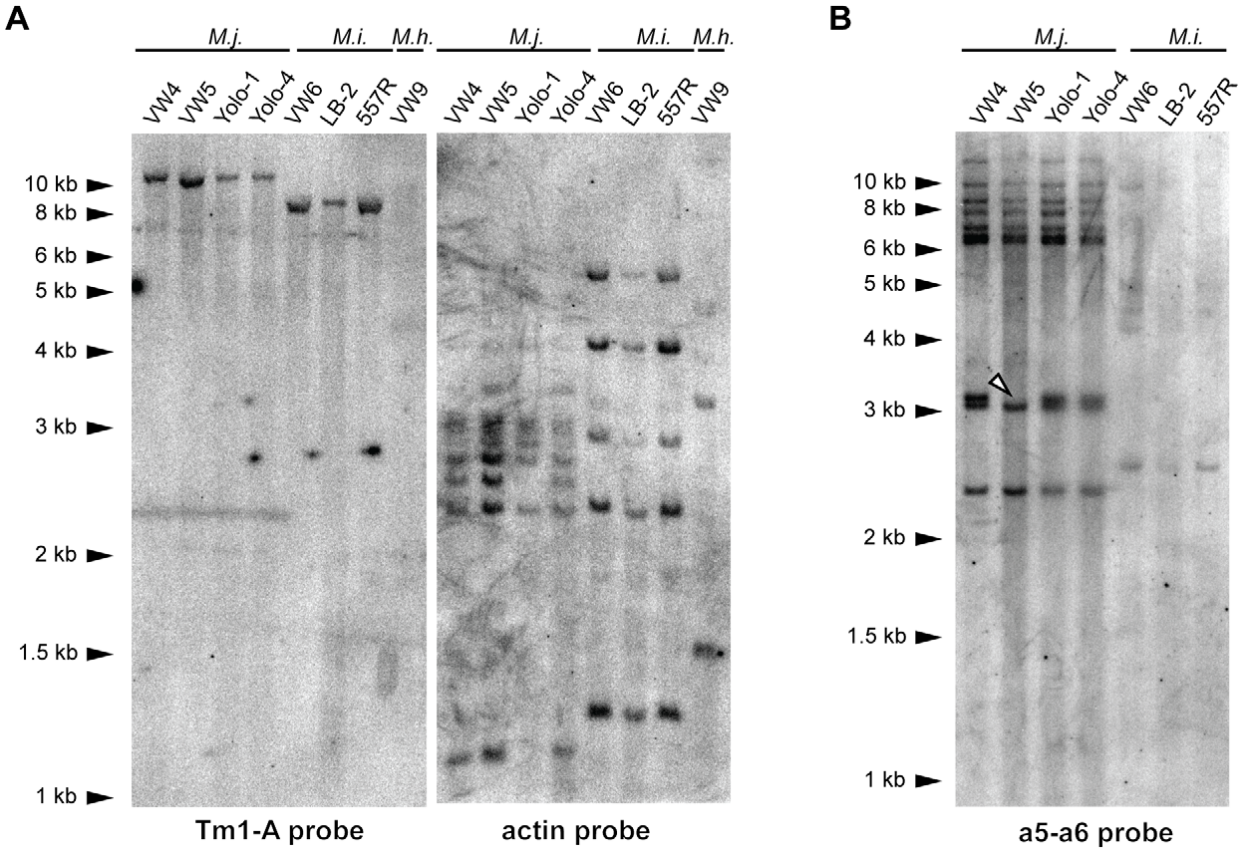
Tm1 elements are low copy and do not display recent transposition. (A) A Southern blot of *Hin*dIII-digested genomic DNA from indicated root-knot nematode isolates probed with Tm1-A and actin. (B) Similar *Hin*dIII-digested blot probed with the a5–a6 probe from *Cg-1*. The position of the missing Tm1 element in VW5 is marked with an arrowhead). Probes are as diagrammed in Figures 1 (a5–a6) and 2 (Tm1-A).

To determine the copy number of Tm1-A elements, a Southern blot of *Hin*dIII-digested genomic DNA from several isolates representing three RKN species was probed with an internal portion of the Tm1-A element (Figure 2B). All surveyed *M. javanica* isolates have a single ∼11 kb hybridizing *Hin*dIII fragment, while all surveyed *M. incognita* isolates have a single ∼8 kb hybridizing fragment (Figure 3A). No *Hin*dIII fragments hybridizing to the Tm1-A probe were detected in *M. hapla* VW9, consistent with PCR results (above) and bioinformatic analyses (discussed below). The same blot was stripped and re-probed with a fragment of beta actin (GenBank AF532605; Materials and Methods). The beta actin hybridization patterns were also similar between isolates of the same species, although *M. javanica* Yolo-1 lacked hybridizing fragments of approximately 2.5 and 1.25 kb when compared to other *M. javanica* isolates. A Southern blot of *M. javanica* VW4 genomic DNA cut with *Bam*HI, *Xba*I, or *Xho*I and probed with Tm1-A also revealed only a single hybridizing fragment (13 kb, 12 kb, 11 kb, respectively; data not shown). These results are consistent with the presence of a single copy of Tm1-A in the genome of tested *M. javanica* and *M. incognita* isolates, and the absence of a conserved copy in *M. hapla* VW9. Furthermore, the similar size of the *Hin*dIII fragments suggests this element is likely in the same genomic location within the isolates of each species.

To determine whether Tm1-HH elements like that at *Cg-1* are in distinct genomic positions between isolates, a portion of the element at *Cg-1* (Figure 1A) [24] was used to probe a second Southern blot of *Hin*dIII-digested genomic DNA (Figure 3B). With the exception of the previously noted absence of a 3.1 kb hybridizing fragment in *M. javanica* VW5 representing the deletion of *Cg-1* [24], the same pattern of approximately nine strongly hybridizing bands was seen in all *M. javanica* isolates (VW4, Yolo-1, and Yolo-4; Figure 3B). This is in agreement with previous findings obtained by cloning and sequencing PCR products that *M. javanica* has 9 copies of sequences with 87 to 100% identity to *Cg-1* [24]. In contrast, only weakly hybridizing fragments are observed in the *M. incognita* isolates suggesting that any copies present are sufficiently diverged and incapable of hybridizing with the probe at the stringent conditions used. This is consistent with our query of the *M. incognita* genome, which found the Tm1-HH element on contig 1763 to be the most similar sequence (72.4% identity over the length of the probe). The similar hybridization patterns present in the tested *M. javanica* isolates suggest that like Tm1-A, non-autonomous Tm1-HH elements have not been mobilized since these isolates diverged.

### Bioinformatic comparison of Tm1 elements in *M. incognita* and *M. hapla*

We sought to identify additional Tm1 elements in the published genomes of *M. incognita* and *M. hapla* using a bioinformatics approach. In short, we used a BLASTN-based algorithm to search for two or more matches on the same contiguous sequence with similarity to a 38 bp region within the Tm1 TIRs in an inverted orientation (Materials and Methods). This method identified 39 elements in *M. incognita* and 22 elements in *M. hapla*, ranging in length from 0.17 to 2.5 kb (Tables S1 and S2; summarized in Table 2).

**Table 2:** Summary of Tm1 elements in *M. incognita* and *M. hapla*.

Tm1 Class	Description	*M. incognita*	*M. hapla*	Average Length
Tm1-A	Putative autonomous element	1	0	2593
Tm1-D	Deletion derivatives of Tm1-A	0	8	1307
Tm1-HH	Contains histone haripin element	8	0	1182
Tm1-ML	MITE-Like	30	12	431
Other	No resemblance to other Tm1 elements	0	2	788
**Total**		**39**	**22**	

Summary of Tm1 element classes and their average lengths in *M. incognita* and *M. hapla*. Detailed descriptions of each element can be found in appendix Tables S1 to S4.

Tm1 elements of *M. incognita* could be divided into three categories based upon features of sequences between the TIRs (Table S1). Only 1 transposase-encoding element, on contig 274 as previously noted, was identified. Eight Tm1 elements, including the aforementioned element on contig 1763, included sequence that matched or resembled the histone hairpin of the *Cg-1* element. The region with similarity to the *Cg-1* element varied, but generally spanned the ∼24 nt histone hairpin and 300–400 nt 3′ of this sequence, in some cases extending to the TIR. We refer to these elements as the histone-hairpin class (Tm1-HH). Tm1-HH elements are about 900 bp except for two, including the previously described element on contig 1763, which contain insertions (Table S1). Upstream of the histone hairpin region, elements in this group vary in their similarity to *Cg-1*, with the element of contig 1763 being the most similar and some other elements showing no similarity. The third category of Tm1 elements is composed of elements typically less than 0.5 kb (with the exception of an element on *Mi* contig 3754, which contains a nested 878 bp inverted repeat sequence). Elements in this third class have inverted repeat sequences between their TIRs, thus resembling MITEs (miniature inverted-repeat transposable elements [42]). These MITE-like Tm1 (Tm1-ML) elements lack Motif D (Table S1) and thus do not have the SG6 primer-binding site within their TIRs, accounting for our failure to detect them by PCR. The Tm1-ML elements are the largest category with 30 of 39 copies identified in *M. incognita* and share 85.9% sequence identity as a group.

No elements capable of encoding an intact transposase were identified in *M. hapla*, but 8 elements with short patches of similarity mainly spanning approximately 200 to 600 bp 3′ to the MULE domain of the Tm1A transposase gene were noted (Table S2). These 8 *M. hapla* Tm1 elements appear to be derivatives of Tm1-A (hereafter called Tm1-D elements). None of these elements retained any homology to the MULE domain, and, aside from portions sharing similarity to the Tm1-A element, share only 52.3% pairwise sequence identity as a group. Queries of the *M. hapla* genome with both the nucleotide sequence and protein sequence of the Tm1-A element failed to detect full-length contiguous sequences, further suggesting *M. hapla* does not have an autonomous Tm1-A element.

No elements resembling the Tm1-HH class were found in *M. hapla*. Like *M. incognita*, the largest category of Tm1 elements in *M. hapla* (12 of 22) is Tm1-ML elements, sharing 90.3% sequence identity as a group. As with the ML elements of *M. incognita*, those of *M. hapla* lack Motif D in their TIRs (Table S2). The Tm1-ML element class is thus the only one clearly shared between *M. incognita* and *M. hapla*, though a neighbor-joining consensus tree generated from an alignment of Tm1-ML elements demonstrates the *M. hapla* elements are more closely related to each other than the majority of *M. incognita* elements (Figure S1). Two elements in *M. hapla* (on contigs 402 and 53) contain internal sequences that did not fit within the Tm1-A, -D, -HH, or -ML classes (noted as “other” in Table S2).

None of the non-autonomous Tm1 elements have significant coding potential, and BLASTX fails to detect similarity to known or predicted proteins in the GenBank nr (non-redundant) database (all E values >1). Similarly, conceptual translations of predicted genes and ORFs residing within Tm1 elements produce only short peptides not significantly similar to known or predicted proteins (all E values >1; data not shown).

### Tm1 TIRs have both conserved and variable motifs

**Figure 4:**
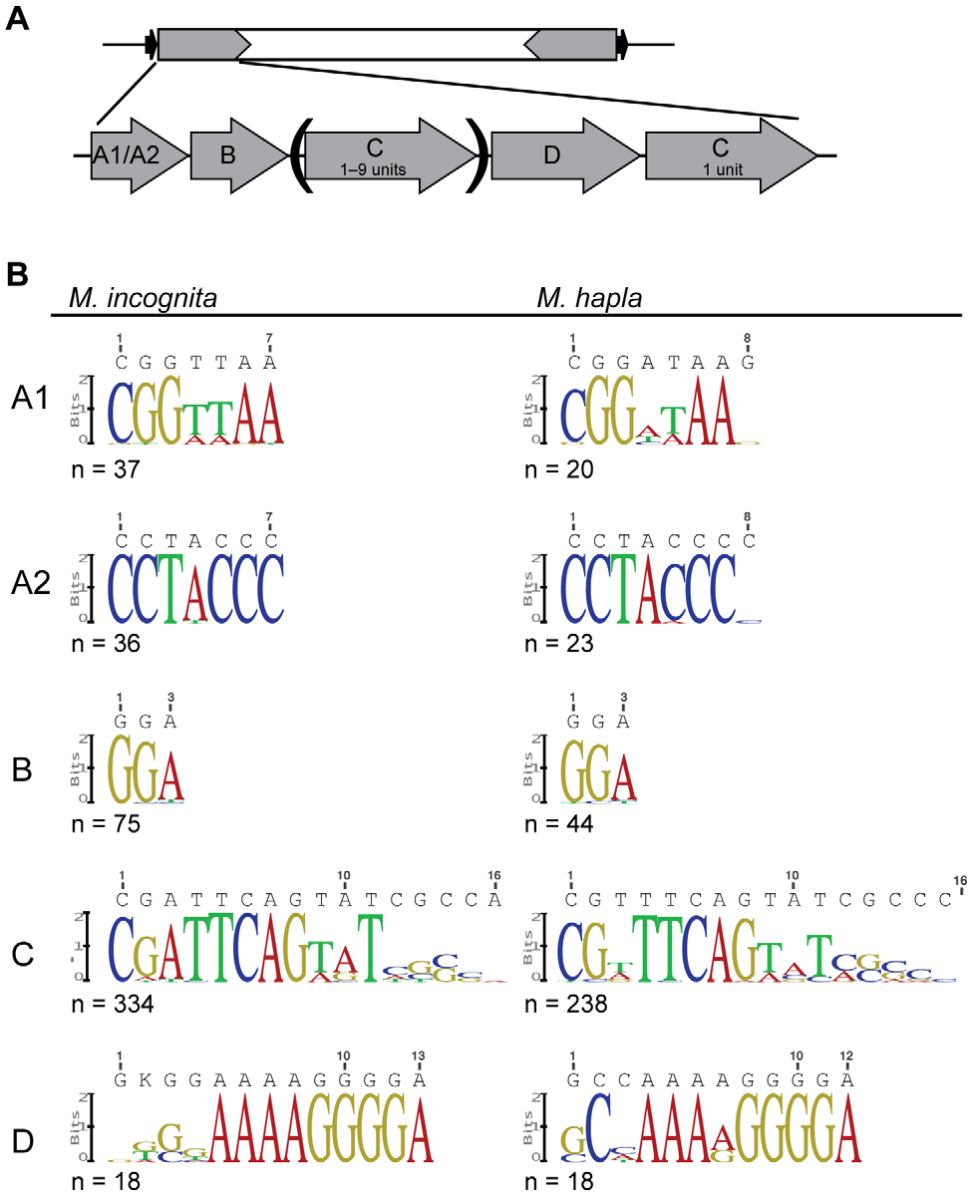
Motifs in TIRs of Tm1 elements. (A) Diagram showing the TIR motif structure of a generic Tm1 element. A1 is generally present at one terminus and A2 at the other. (B) Sequence logos demonstrating variability of Tm1 TIR motifs in *M. incognita* and *M. hapla*. Sequence logos and the associated consensus sequence are only shown for positions where the bit score is greater than 0.

The TIRs of Tm1 elements annotated in the *M. incognita* and *M. hapla* genomes resemble the TIRs of Tm1 elements described previously (Figure 1A, 2B), but have some differences in the composition and sequence of motifs (Figure 4A). Most Tm1 elements identified in *M. incognita* (24/39) and *M. hapla* (20/22) have the 7 bp A1 Motif exterior to the TIR at one end and A2 at the other end of the element (Tables S3 and S4). However, for 6 elements, only one terminal motif was detected, and in one case (Tm1-ML on *M. hapla* contig 1532) Motif A2 was found on both ends of the element. Motif A2 (5′-CCTACCC-3′) is highly conserved in both *M. incognita* and *M. hapla* Tm1 elements (Figure 4B). However, Motif A1 displays some variability (Figure 4B), with a consensus of 5′-CGGTTAA-3′ in *M. incognita*, and 5′-CGGATAA-3′ in *M. hapla*. Most Tm1 elements have a 3 bp Motif B internal to Motifs A1 and A2 with a clear 5′-GGA-3′ consensus in both *M. incognita* and *M. hapla*.

Tm1 TIRs differ in length due to the variable number of Motif C units (Figure 4A). Tm1 TIRs contain 1–9 consecutive units of Motif C, and differences in the number of Motif C repeats occur both between elements and for opposite ends of the same element (Tables S1 and S2). In total, the 39 *M. incognita* elements contain 334 units of Motif C within their TIRs, ranging in length from 8 to 17 bp with the most common length being 14 bp (191 of 334). Similarly, within the 22 *M. hapla* elements, there are 238 Motif C units with lengths of 12 to 21 bp, but most commonly 14 bp (144 of 238). Sequence logos demonstrate a core consensus sequence of Motif C (5′-CGATTCAGTATCGC-3′ for *M. incognita*; 5′-CGTTTCAGTATCGC-3′ for *M. hapla*) with repeats more highly conserved at their 5′ ends than the 3′ ends (Figure 4B).

While Tm1-ML elements lack the GA-rich Motif D, this motif is generally present and highly conserved in other Tm1 elements (Tables S1 and S2). The sequence consensus of Motif D differs between *M. incognita* (5′-GKGGAAAAGGGGA -3′; where K = G or T) and *M. hapla* (5′-GCCAAAAGGGGA -3′). In both species, the 5′ end of Motif D is less conserved than is the 3′ end (Figure 4B). The SG6 primer binding site spans part of the variable portions of Motif D (Figure 1B), accounting for our inability to detect all Tm1 elements in *M. javanica*, *M. incognita*, and *M. hapla* via PCR.

In addition to differences in motif composition, the TIRs of each element differ in sequence. BLAST2 was used to identify local regions of similarity between the two TIRs of each element, allowing automatic exclusion of asymmetric regions such as Motif A1, A2, and asymmetric numbers of Motif C units. Subsequently, the BLAST2 identified regions were aligned for comparison. Of the 61 elements analyzed, only 5 elements (Tables S1 and S2) display 100% sequence identity between the BLAST2-defined portions of their TIRs. On average, the two TIRs of a single element are 93.15% identical due to SNPs and small indels.

### Tm1 elements are commonly flanked by 9 bp target site duplications

Analysis of complete Tm1 elements having both terminal motifs A1 and A2 revealed that in most cases (45 of 53), elements are flanked by 8–10 bp (though most commonly 9 bp) target site duplications (Tables S3 and S4). Comparison of TSD sequences did not reveal a clear consensus sequence, suggesting Tm1 elements do not have target sequence preferences at their integration site.

## Discussion

A deletion in the *M. javanica* genome encompassing a candidate effector gene led us to the discovery of a novel transposable element family that we have named Tm1. These elements are defined by the shared novel structure and sequence of their TIRs, which contain 4 sequence domains and varying numbers of an internally redundant ∼14 bp sequence motif and, thus, resemble *Foldback* (FB) elements, a heterogeneous group of transposons with composite TIRs of multiple domains and tandem repeats [43], [44], [45], [46]. Tm1 elements contain at their boundaries asymmetric 7 bp terminal motifs, generally Motif A1 at one end of the element and Motif A2 at the other, that are outside of the inverted repeat. The presence of short, non-inverted sequences at the ends of elements is unusual, but has been observed in other *Mutator*-like elements, which are thus sometimes said to have sub-terminal inverted repeats (sub-TIRs) [36], [47]. Additional conserved motifs characterizing Tm1 elements include a 3 bp Motif B and a GA-rich motif D, which is generally followed by a unit of Motif C.

Based on bioinformatic analysis of available genomic sequence, *M. incognita* Tm1 elements can be placed in three general categories: Tm1-A, Tm1-HH, and Tm1-ML. The isolates of *M. incognita* and *M. javanica* that we examined carry a single Tm1-A element encoding a protein resembling a *Mutator*-like (MULE) transposase. The presence of 9 bp TSDs flanking most Tm1 transposons is consistent with the size commonly generated by MULE domain transposases [47], [48], [49] and other members of the IS256/MULE transposon family [50], [51], [52], [53]. The domain structure of the putative transposase places Tm1-A and the Tm1 family within the *Phantom* subclass of *Mutator* elements with highest similarity to sequences from insects [36]. The predicted transposases of *Phantom* elements are characterized by an N-terminal, FLYWCH zinc-finger DNA binding domain and a MULE transposase domain. It is unclear whether the Tm1-A transposase can bind DNA as it lacks the final histidine residue to form a complete C2H2 zinc-finger motif within the FLYWCH domain. However, it is possible Tm1-A can localize to DNA in a manner similar to the *Drosophila* FLYWCH domain isoforms of Mod(mdg4) proteins, which do not bind DNA directly but modulate gene expression through interactions with DNA binding proteins [37], [54], [55]. Further biochemical analysis of the Tm1-A-encoded protein is needed to clarify any function.

Recently some *Foldback* elements have been grouped with *Mutator* transposons into a superfamily because of similarity between their encoded transposases [56]. Our finding of a *Phantom*-like transposase within *FB*-like TIRs supports this grouping. However, it is clear that despite their structural similarity, *FB*-like elements are not necessarily phylogenetically related as different classes of transposases have been shown to reside within their TIRs and mediate their transposition. For example, the *FB* transposon *Galileo* in *Drosophila* harbors a P element transposase [57] whereas the Arabidopsis *FB* element *FARE* contains a MULE domain transposase [46]. In addition, while some *Phantom* transposases are flanked by *FB*-like TIRs or sub-TIRs, others are flanked by structurally simpler TIRs more common in the *Mutator* superfamily [36]. Such observations have led others [36], [57] to propose that *FB*-like transposon families evolved independently from structurally simple transposons through the gradual expansion of TIR length. A similar case of TIR expansion toward a *FB*-like structure appears to have occurred during the evolution of the RKN *Meloidogyne chitwoodi* Tc1/*Mariner*-like element *Mcmar*, which has 355 bp TIRs composed of direct repeats and palindromes unlike most Tc1/*Mariner* elements, which have simple 26–30 bp TIRs [58].

We hypothesize the non-autonomous Tm1 elements are derived from an ancestral autonomous Tm1-A element, in a manner consistent with models of transposon family evolution [59]. For example, Pack-MULES are derivatives of plant *Mutator* transposons that have acquired fragments of host genes [60], [61]. Tm-1-HH elements, characterized by the presence of a highly conserved histone hairpin motif, may represent a similar acquisition of host sequences by a Tm1 element. Prior to the discovery of *Cg-1* [24], histone-hairpin motif had been found only in the 3′ UTR of transcripts of replication-dependent histone genes in metazoans. The highly-conserved 24-nt hairpin and sequences 3′ serve as binding sites for an elaborate processing machinery that produces a specific endonucleolytic cleavage of the mRNA just 3′ of the hairpin. As a result of this cleavage, these histone mRNAs are not polyadenylated [29]. Interestingly, the region of the Tm-1-HH element that is most conserved among this group is 3′ of the histone hairpin structure, that is, the 3′ end of the transcript that is predicted to be cleaved off by hairpin processing machinery. Further studies will be required to determine if the histone hairpin region is under selection and if RNAs produced by Tm1-HH elements have a function.

Transposable elements can be acquired through horizontal gene transfer (HGT; for reviews see [62], [63], [64]). However, despite the similarity of the Tm1-A transposase to insect *Phantom* transposases (Table 1), Tm1-A lacks sufficient DNA sequence conservation with these elements to demonstrate HGT as has been done for other transposons [65], [66], [67], [68]. A more parsimonious assumption is that the Tm1 elements described here evolved from a functional Tm1-A-like element present in the ancestral *Meloidogyne* species rather than a recent horizontal gene transfer event from insects. Though firm conclusions cannot be drawn from current data, our observations are consistent with a model in which the Tm1-HH and Tm1-D classes arose as distinct deletion/insertion derivatives of Tm1-A in the *M. incognita* group and *M. hapla* lineages, respectively. Additionally, though the Tm1-ML elements are structurally similar between *M. incognita* and *M. hapla*, most show evidence of having evolved independently as deletion derivatives of larger Tm1 elements in each lineage: the majority of (20 of 30) *M. incognita* Tm1-ML elements have a *M. incognita*-type domain A1 (CGGTTAA; Figure 4) and the majority of (9 of 12) *M. hapla* Tm1-ML elements have a *M. hapla*-type domain A1 (CGGATAA; Figure 4). Additionally, the *M. hapla* Tm1-ML elements form a well-supported clade distinct from most *M. incognita* Tm1-ML elements (Figure S1).

Our data is consistent with Tm1 elements having been mobile during the evolution of root-knot nematode species. The presence of an additional 8 Tm1-HH elements similar in sequence to that at *Cg-1* in *M. javanica* but not in *M. incognita* (Figure 3B) suggests that this particular element has expanded in the *M. javanica* lineage or lost in the *M. incognita* lineage. The presence of an insertion in the site flanked by primers 17-R and 17-F in *M. javanica*, but an empty site only in *M. incognita* suggests that this may be a relatively recent transposon insertion site (Fig. 1C). However, our Southern analyses also suggest neither Tm1-A or Tm1-HH elements have experienced recent transposition activity. Each isolate of *M. javanica* and *M. incognita* has a species-specific, rather than isolate-specific, hybridization pattern with the Tm1 probes (Figure 3). In fact, our Southern analysis indicates that the beta actin genes of *Meloidogyne* are more polymorphic than Tm1 elements (Figure 3A). It is important to note that most nematode isolates tested in this study (with the exception of *M. incognita* 557R, which was derived from a field collection in North Carolina) were collected from different locations in the California Central Valley and a wider sampling may be necessary to reveal rare Tm1 transposition events.

Consistent with the molecular observations suggesting recent Tm1 element inactivity, bioinformatically-detected Tm1 elements in the *M. incognita* and *M. hapla* genomes display sequence features consistent with senescence and deterioration. As Class II transposon families age, the number of autonomous elements decreases while the number of non-autonomous members increase and diverge in sequence [59], [69]. Moreover, as transposable elements are commonly deleterious, asexual lineages are predicted to contain largely inactive and decaying transposable elements [70]. Consistent with these hypotheses, tested *M. javanica* and *M. incognita* isolates have retained only a single putatively autonomous Tm1-A element while *M. hapla* encodes no elements capable of encoding a transposase, and the majority of the Tm1 family in both *M. incognita* and *M. hapla* is composed of MITE-like elements. Furthermore, no two elements are identical in sequence, suggesting they have accumulated mutations, including insertions, since the expansion of the Tm1 family. Additionally, some Tm1 elements are missing TIR motifs, differ in copy number of Motif C repeats, or lack TSDs. One Tm1 element in *M. hapla* (contig 1532) has Motif A2 at the end of both TIRs and lacks a TSD, and may have arisen as a crossover event between two Tm1 elements. Moreover, the Tm1-HH element at *Cg-1* appears to be degenerate and lacks terminal Motif A2 (Figure 1A), possibly due to an intra or inter-element recombination event (Figure 1A). Together these observations support that Tm1 elements represent a currently inactive and senescent transposon family in root-knot nematodes.

The role of the loss of the Tm1-HH element at the *Cg-1* locus in the acquisition of ability to bypass resistance in tomato mediated by the *Mi-1* gene remains unclear. Current models based on similar plant resistance genes predict that the Mi-1 protein either directly or indirectly recognizes the presence of a nematode product (elicitor) to trigger defense responses [71]. Previous results [24] suggest the virulent *M. javanica* VW5 strain is missing the elicitor that is present in its progenitor strain VW4 and support the importance of the *Cg-1* transcript for Mi-1-mediated resistance. However, several Tm1-HH elements similar to that at *Cg-1* are present in both strains VW4 and VW5, and DNA blots showed that *Cg-1* is present in the virulent *M. javanica* field isolates Yolo-1 and Yolo-4 (Figure 3 and [24]). It may be that the Tm1-HH element carrying *Cg-1* regulates expression of a *cis*-linked nematode gene that is responsible for triggering resistance. Transposable elements contain enhancers and promoters, often in their TIRs, and thus can act as regulators of *cis*-linked genes (reviewed in [13]). RNAi silencing of *Cg-1* may reduce or eliminate expression of the *cis*-linked gene by, for example, modifying chromatin structure in the region. In the case of VW5, the deletion of *Cg-1* extends at least 2 kb 5′ of the Tm1-HH element (S. Gross and V. Williamson, unpublished observations) and, thus, the *cis*-linked elicitor gene may be deleted. However, these ideas remain untested as the extent of the deletion has not been determined and a *cis*-linked effector has not yet been identified.

Given the presence of multiple Tm1-HH elements in our virulent and avirulent isolates, the truncated nature of the Tm1-HH element at *Cg-1*, data suggesting the deletion extends beyond the Tm1 element at *Cg-1*, and the apparent inactivity of extant Tm1 elements, it is unlikely that canonical cut-and-paste transposition of Tm1 caused deletion of *Cg-1* in VW5. However, other rearrangements involving the Tm1-HH element at *Cg-1* may have occurred, such as ectopic recombination and chromosome breakage [72], [73]. In fact, internally repetitive TIRs of *Foldback* elements appear to be hotspots for ectopic recombination, as has been shown in studies of chromosome rearrangements in natural populations of *Drosophila* [74], [75]. In asexual lineages, which cannot acquire beneficial alleles that may arise in other members of the population, recombination between repetitive sequences leading to genome alterations may prove advantageous. Similar to Barbara McClintock's “Genome Shock” theory [76], asexual RKN parasites may use genomic change to their advantage when faced with the need to surmount plant resistance genes or perish. Such a mechanism has been proposed for some pathogenic bacteria, where effector molecules are clustered in “pathogenicity islands” flanked by transposable and repetitive elements and readily altered to avoid host defense responses [77], [78]. Perhaps because its asexual reproduction mode benefits from use of repetitive sequences for adaptation, the genome of *M. incognita* has much more repetitive DNA than that of the sexual species *M. hapla* [10], [11]. Whether Tm1-mediated genomic alterations produced the deletion of an effector molecule that allowed this nematode strain to bypass recognition by the tomato resistance gene product remains to be determined. However, such activity is consistent with hypotheses that transposons and repetitive elements contribute to creating genetic diversity in asexual organisms such as the root-knot nematodes *M. javanica* and *M. incognita* [11], [12], and may represent an example of transposable element exaptation [13], [79].

## Materials and Methods

### Collection and culture of nematodes

*M. javanica* nematode strains VW4 and VW5 [24]; *M. incognita* strain VW6 [80] and *M. hapla* strain VW9 [21], [31], [81] have been described previously. Additional isolates (*M. javanica* isolates Yolo-1, Yolo-2, and Yolo-4 from Yolo County, CA; *M. incognita* isolates Los Banos-2 from Merced County, CA and W-1 from Yolo County, CA) were obtained from processing tomato fields. *Meloidogyne incognita* strain 557R was provided by A.C. Triantaphyllou, North Carolina State University. Nematodes were identified to species by PCR using species-specific and mitochondrial primers [82], [83]. Nematodes were maintained on tomato cultivars VFNT (*Mi/Mi*) or UC82 (*mi/mi*). Eggs and second-stage juveniles (J2s) were collected as described previously [84] then frozen in liquid nitrogen and stored at −80°C.

### Molecular analyses

To prepare DNA, 200–300 µl of packed frozen eggs were pulverized in a mortar and pestle with liquid nitrogen. An equal volume of homogenization buffer (200 mM NaCl, 200 mM Tris pH 7.5, 20 mM EDTA, 2% SDS, 0.04 M 2-mercaptoethanol) was added to the mortar and grinding continued until eggs were completely homogenized. The homogenate was incubated at 40°C for 30 minutes with 0.2 mg/ml proteinase K. Homogenate was extracted using an equal volume of a 1∶1 mixture of phenol∶chloroform, then repeated with chloroform only. DNA was precipitated with 2 volumes ethanol and 0.1 volumes 3 M sodium acetate, washed in 70% ethanol, and resuspended in TE (10 mM Tris pH 8.0, 1 mM EDTA). Phenol∶chloroform extraction and ethanol precipitation were repeated, and the DNA was resuspended in TE, treated with RNAse A, and quantified using a fluorometer. Prior to all PCR reactions, DNA was diluted to 5 ng/µl in TE.

Primer sequences used in this work are listed in Table 3. Amplification conditions for primer sets: SG1+SG6: 35 cycles of 95°C—30 s, 58°C—30 s, 72°—60 s; 17-F+17-R: of 95°C—30 s, 60°C—30 s, 72°C—75 sec; 17-R+SG2: 95°C—30 s, 58°C—30 s, 72°C—30 sec. Reactions were performed according to the manufacturer's guidelines with *Taq* polymerase (New England Biolabs, Ipswich, MA) in 1× Thermo Pol buffer supplemented with MgCl_2_ to a final concentration of 1 mM. PCR reactions with the single primer SG6 were performed using LongAmp Polymerase (New England Biolabs, Ipswich, MA) according to the manufacturer's guidelines in buffer supplemented with KCl to a final concentration of 30 mM. Cycling conditions were 35 cycles of 95°C—30 s, 58°C—30 s, 65°C—8 min. All separations of PCR amplicons were performed in 1% LE agarose, 1× TBE gels. PCR amplicons were cloned into pGEM-T Easy Vector (Promega, Madison, WI) and transformed into *E. coli* XL1-Blue (Stratagene, La Jolla, CA) according to the manufacturer's instructions. The SG1+SG6 and 17-F+17-R PCR amplicons were sequenced directly; SG6 amplicons were sequenced from plasmid clones. The UC Davis College of Biological Science DNA Sequencing Facility performed all sequencing services.

**Table 3:** Primers used in this study.

Name	Sequence (5′ to 3′)	GenBank Accession(s)	Reference
a5	GAGCCGTCCATTTTAAACCA	EU214531.2	[24]
a6	GGGTTAAGGTTGTTGTTGCC	EU214531.2	[24]
SG1	CGAGAATTCTACACTGACAATG	EU214531.2	This work
SG2	ACTGAATCGTCCCCTTTTCC	HQ122410.1	This work
SG6	GATTCAGTATACGGGGAAAAGG	EU214531.2; HM470230.1–HM470232.1	This work
M8-1F	TGGCTTTCTATATGTTTTTCATGC	HM470230.1–HM470232.1	This work
M8-3R	GTAAGTTGCTGTCAGTGCAAGG	HM470230.1–HM470232.1	This work
M8-6R	TCAGAATCTGCCAAAAGAAACC	HM470230.1–HM470232.1	This work
MjActin RT-F	AAGCCGTTCTTTCTTTGTATGC	AF532604.1	[24]
MjActin RT-R	AAGAATAACCACGTTCAGTGAGG	AF532604.1	[24]
Mj bActin-F	TAGGTATGTTGCCATCCAAGC	AF532604.1	This work
Mj bActin-R	CAAAGCAGTAATTTCCTTCTGC	AF532604.1	This work
17-F	AGAGCTCGGGACTGAAACGTCC	HQ122410.1	This work
17-R	TCTCCCTCGCCTCATCTCCACG	HQ122410.1	This work

RNA was isolated from approximately 200–300 µl of packed nematode eggs using Trizol reagent (Invitrogen, Carlsbad, CA). Reverse transcription was performed with 5 µg DNAse I-treated RNA using SuperScript III (Invitrogen) and oligo(dT)_12–18_ (Invitrogen) according to the manufacturer's directions in a total volume of 20 µl. One µl of resulting cDNA was used per 25 µl PCR reaction with NEB Long Amp polymerase (as above) for the following conditions the primer pair M8-1F+M8-3R, 50 cycles of 95°C—30 s, 58°C—30 s, 65°C—80 s. As a positive control, beta actin was amplified from cDNA using Taq polymerase and primers Mj-Actin-RT-F and Mj-Actin-RT-R for *M. javanica* samples (35 cycles of 95°C—30 sec., 58°C—30 sec., 72°C—30 sec.). Amplicons were sequenced directly as described above.

Restriction digests of either 5 or 10 µg of genomic DNA were performed according to the restriction enzyme manufacturer's directions (New England Biolabs). DNA was separated in 1.0% LE agarose and 1× TBE and blotted to Hybond-N nylon membranes (GE Healthcare Life Sciences, Piscataway, NJ) following standard protocols [85]. Tm1-A probe templates were synthesized from a M8-1F and M8-6R PCR amplicon generated from a plasmid containing a 2.3 kb SG6 amplicon from VW4. Template for Tm1-HH probe was generated by amplification with primers a5 and a6 from a plasmid containing a cloned VW4 a5-a6 amplicon [24]. Beta actin probe was amplified from *M. javanica* VW4 genomic DNA template using primers Mj bActin-F and Mj bActin-R (95°C—30 s; 58°C—30 sec, 72°C—1 min). Probe templates were isolated from 1% TBE gels using a QIAquick Gel Extraction Kit (Qiagen, Valencia, CA). Randomly-primed probes were labeled with [α^32^P]-dATP (Perkin Elmer, Waltham, MA) according to published protocols [86]. Blots were hybridized in aqueous buffer at 60°C following standard protocols [85].

A plasmid library of ∼3 kb *Hin*dIII fragments of *M. javanica* VW4 genomic DNA was constructed as follows: 50 µg genomic DNA was digested with *Hin*dIII and separated on a 0.8% LE agarose gel in 1× TBE. Four size fractions spanning the range of 2–4 kb were isolated from the gel, and DNA was extracted from these fractions using a QiaQuick Gel Extraction Kit (Qiagen, Valencia CA). The four fractions of gel-extracted DNA were amplified using primers SG1 and SG6 to identify the fraction containing the highest amount of *Cg-1* PCR template. The *Hin*dIII fragments were cloned into Litmus38i (New England Biolabs) that had been previously treated with alkaline phosphatase. Approximately 10,000 independent clones were screened via PCR using pooling methods described previously [87].

A BAC library of *M. javanica* VW4 DNA was prepared with high molecular weight DNA isolated from J2s by Lucigen Corporation (Middleton, WI). A total of 12,672 BAC clones with an average insert size of 50 kb was screened via PCR with SG1 and SG6 primers using PCR conditions as above but with the annealing temperature lowered from 58°C to 54°C. The identified BAC clone (BAC 18M17) was isolated and purified according to the instructions provided by Lucigen. Sequencing and sequence assembly of BAC 18M17 was completed by the UC Davis College of Agricultural and Environmental Science Genomics Facility.

### Genbank deposits

The following sequences were deposited at GenBank: *M. incognita* Los Banos-2 Tm1-A, HM470230.1; *M. javanica* VW4 Tm1-A, HM470231.1; *M. javanica* Yolo-1 Tm1-A, HM470232.1; *M. javanica* VW4 BAC 18M17, HQ122410.1; *M. javanica* VW4 0.4 kb 17-F+17-R amplicon, HQ122411.2. The original sequence of the *M. javanica Cg-1* locus (EU214531.1) was updated and corrected with the sequence of the 3.1 kb *Hin*dIII clone, EU214531.2.

### Bioinformatic analyses

The genome of *Meloidogyne incognita* (GenBank accessions CABB01000001–CABB01009538) [10] was searched using BLASTN [88] for sequences homologous to *Cg-1* (GenBank EU214531). Additional elements flanked by similar inverted repeats were identified using BLASTN to search the genomes of *M. incognita* and *M. hapla* [31] (GenBank accessions ABLG01000001–ABLG01003450) for sequences similar to a 38 bp sequence in the inverted repeat flanking *Cg-1* (5′–GATTCAGTAT ACGGGGAAAA GGGGACGATT CAGTATAC–3′) with the following parameters (word size = 7; low-complexity filter = on; max E value = 10; gap costs: open = 4, extend = 2; scoring: match = 1, mismatch = −3). Using this method, we obtained a total of 142 BLASTN hits in *M. incognita*, and 147 BLASTN hits in *M. hapla*. Two or more BLASTN hits residing on the same contig in opposite orientation relative to each other suggested presence of an intact Tm1 element. A BLAST2 analysis [89] with the following parameters (word size = 11, complexity filter = on, match = 1, mismatch = −2, open gap penalty = 5, gap extension penalty = 2, gap×dropoff = 50, E-value cutoff = 10) was performed to clarify the full extent of the terminal inverted repeats. Tandem repeats of Motif C were annotated manually. Sequence alignments were generated using Geneious v4.6 [90]. Tandem repeats were aligned using ClustalW v1.82 [91] in conjunction with Geneious v4.6 with the following parameters: free end gaps allowed, gap open cost = 15; gap extension cost = 6.66. Sequence logos [92] were generated using Geneious v4.6. Global alignments with free end gaps of Tm1 elements were done using Geneious Aligner, part of Geneious v4.6, with the following parameters: Cost matrix: 65%, match = 5, mismatch = −4, open gap penalty = 12, gap extension penalty = 3.

Predicted protein sequences were analyzed using Pfam v. 24.0 [32]. Nuclear localization signals were identified using PSORT II [40]. GeneMark-ES [93], [94] was used to predict putative genes, using the *C. elegans* ES-3.0 model for gene prediction parameters.

## Supporting Information

Figure S1 **Unrooted neighbor-joining consensus tree demonstrating the relationship between** ***M. hapla*** **(red) and** ***M. incognita*** **(blue) Tm1-ML elements.** Branch bootstrap support is noted on branches. Tree was generated using Geneious Aligner and Geneious Tree Builder (Drummond *et al.* 2008). Branch lengths were transformed to uniform length for clarity.
(TIF)

Table S1 **Tm1 elements of** ***Meloidogyne incognita*** **.** Target site duplication (TSD) length is indicated if both terminal motifs A1 and A2 are present; n.a. (not analyzed) indicates one or both of these motifs is missing; n.f. (not found) indicates that neither motif A1 or A2 was found. GenBank accession numbers, location on contig, TSD sequences, and sequences of 7 bp terminal motifs are provided in Table S3. When possible, elements are oriented with the Left TIR beginning with Motif A1 and the Right TIR terminating with Motif A2. Letters in parenthesis denote Tm1 elements on the same contig. Notes: ^a^ Identity between TIRs excluding motif A1 or A2. Tm1 elements with nested, uncharacterized transposable elements are noted as follows: ^b^ Contains 937 bp insertion flanked by 9 bp TSD, ^c^ Contains 923 bp insertion flanked by 7 bp TSD, ^d^ contains 877 bp insertion lacking TSD.
(DOCX)

Table S2 **Tm1 elements of** ***Meloidogyne hapla*** **.** TSD length is indicated if both terminal motifs A1 and A2 are present; n.a. (not analyzed) indicates one or both of these motifs is missing; n.f. (not found) indicates that neither motif A1 or A2 was found. GenBank accession numbers, location of Tm1 elements, TSD sequences, and 7 bp terminal motif sequences are provided in Table S4. When possible, elements are oriented with the Left TIR beginning with Motif A1 and the Right TIR terminating with Motif A2. Letters in parenthesis denote Tm1 elements on the same contig. Note: ^a^ Identity between TIRs excluding 7 bp terminal motifs.
(DOCX)

Table S3 **Accessory information of Tm1 elements in** ***Meloidogyne incognita*** **.** The 7 bp terminal motifs A1 and A2 are shown in boldface. TSDs are underlined. No flanking sequence is shown if TSDs or terminal motifs are not present. Mismatches within the presumed TSDs are shown as lowercase letters. Letters in parenthesis denote Tm1 elements on the same contig.
(DOCX)

Table S4 **Accessory information of Tm1 elements in** ***Meloidogyne hapla*** **.** The 7 bp terminal motifs A1 and A2 are shown in boldface. TSDs are underlined. No flanking sequence is shown if TSDs or terminal motifs are not present. Mismatches within the presumed TSDs are shown as lowercase letters. Letters in parenthesis denote Tm1 elements on the same contig.
(DOCX)
