# Supplementary figures and images for: Tm1: A Mutator/Foldback Transposable Element Family in Root-Knot Nematodes
Source: PLoS One. 2011 Sep 8;6(9):e24534. doi: 10.1371/journal.pone.0024534 (PMC3169594; doi:10.1371/journal.pone.0024534)

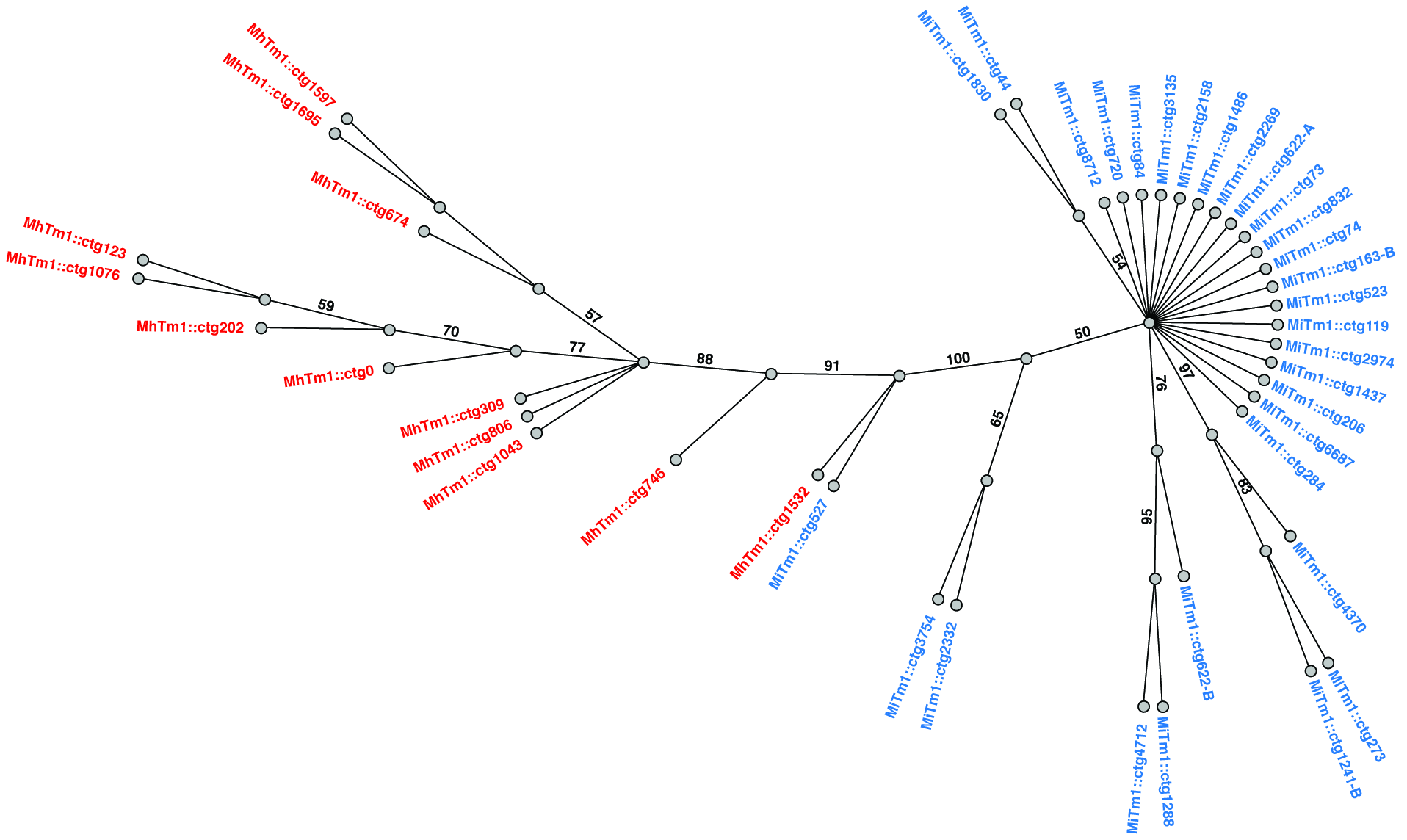

Supplement: Figure S1 — Unrooted neighbor-joining consensus tree demonstrating the relationship between M. hapla (red) and M. incognita (blue) Tm1-ML elements. Branch bootstrap support is noted on branches. Tree was generated using Geneious Aligner and Geneious Tree Builder (Drummond et al. 2008). Branch lengths were transformed to uniform length for clarity. (TIF) [file pone.0024534.s001.tif]
