# Supplementary material for: Tm1: A Mutator/Foldback Transposable Element Family in Root-Knot Nematodes
Source: PLoS One. 2011 Sep 8;6(9):e24534. doi: 10.1371/journal.pone.0024534 (PMC3169594; doi:10.1371/journal.pone.0024534)
Supplement: Table S1 — Tm1 elements of Meloidogyne incognita . Target site duplication (TSD) length is indicated if both terminal motifs A1 and A2 are present; n.a. (not analyzed) indicates one or both of these motifs is missing; n.f. (not found) indicates that neither motif A1 or A2 was found. GenBank accession numbers, location on contig, TSD sequences, and sequences of 7 bp terminal motifs are provided in Table S3. When possible, elements are oriented with the Left TIR beginning with Motif A1 and the Right TIR terminating with Motif A2. Letters in parenthesis denote Tm1 elements on the same contig. Notes: a Identity between TIRs excluding motif A1 or A2. Tm1 elements with nested, uncharacterized transposable elements are noted as follows: b Contains 937 bp insertion flanked by 9 bp TSD, c Contains 923 bp insertion flanked by 7 bp TSD, d contains 877 bp insertion lacking TSD. (DOCX) [file pone.0024534.s002.docx]

**Table S1. Tm1 elements of *Meloidogyne incognita.***

|  |  |  | **Left TIR** | | | | | **Right TIR** | | | | |  |  |
| --- | --- | --- | --- | --- | --- | --- | --- | --- | --- | --- | --- | --- | --- | --- |
| **Contig** | **Element length (bp)** | **TSD length (bp)** | **Length (bp)** | **Terminal 7 bp** | **B** | **C** | **D** | **Length (bp)** | **Terminal 7 bp** | **B** | **C** | **D** | **Identity between TIRs ^a^** | **Class** |
| 274 | 2593 | 9 | 150 | A1 | 1 | 9 | 1 | 152 | A2 | 1 | 9 | 1 | 99.3% | Tm1-A |
| 1081 | 1020 | 3 | 116 | A1 | 1 | 6 | 1 | 158 | A2 | 1 | 9 | 1 | 84.3% | HH |
| 1241 (A) ^b^ | 1970 | 8 | 144 | A1 | 1 | 8 | 1 | 131 | A2 | 1 | 7 | 1 | 88.5% | HH |
| 163 (A) | 1042 | n.a. | 123 | A1 | 1 | 7 | 1 | 24 | n.f. | 0 | 1 | 1 | 95.8% | HH |
| 1763 ^c^ | 1900 | 9 | 121 | A1 | 1 | 7 | 1 | 93 | A2 | 1 | 5 | 1 | 95.3% | HH |
| 1831 | 867 | 9 | 78 | A1 | 1 | 4 | 1 | 166 | A2 | 1 | 10 | 1 | 97.2% | HH |
| 2092 | 893 | 9 | 158 | A1 | 1 | 9 | 1 | 159 | A2 | 1 | 9 | 1 | 88.0% | HH |
| 6020 | 883 | n.a. | 142 | n.f. | 0 | 9 | 1 | 152 | A2 | 1 | 9 | 1 | 90.3% | HH |
| 847 | 877 | 9 | 152 | A1 | 1 | 9 | 1 | 94 | A2 | 1 | 5 | 1 | 94.0% | HH |
| 119 | 425 | 9 | 68 | A1 | 1 | 4 | 0 | 68 | A2 | 1 | 4 | 0 | 91.8% | ML |
| 1241 (B) | 458 | 0 | 35 | A1 | 1 | 2 | 0 | 105 | A2 | 1 | 7 | 0 | 96.4% | ML |
| 1288 | 391 | 0 | 49 | A1 | 1 | 3 | 0 | 47 | A2 | 1 | 3 | 0 | 90.5% | ML |
| 1437 | 440 | 9 | 68 | A1 | 1 | 4 | 0 | 82 | A2 | 1 | 5 | 0 | 93.4% | ML |
| 1486 | 402 | 10 | 54 | A1 | 1 | 3 | 0 | 54 | A2 | 1 | 3 | 0 | 91.5% | ML |
| 163 (B) | 416 | 9 | 68 | A1 | 1 | 4 | 0 | 54 | A2 | 1 | 3 | 0 | 87.2% | ML |
| 1830 | 457 | 9 | 82 | A1 | 1 | 5 | 0 | 82 | A2 | 1 | 5 | 0 | 93.3% | ML |
| 206 | 444 | 9 | 68 | A1 | 1 | 4 | 0 | 82 | A2 | 1 | 5 | 0 | 88.5% | ML |
| 2158 | 402 | 8 | 54 | A1 | 1 | 3 | 0 | 54 | A2 | 1 | 3 | 0 | 97.9% | ML |
| 2269 | 403 | 9 | 54 | A1 | 1 | 3 | 0 | 68 | A2 | 1 | 4 | 0 | 89.4% | ML |
| 2332 | 379 | 9 | 35 | A1 | 1 | 2 | 0 | 35 | A2 | 1 | 2 | 0 | 100.0% | ML |
| 273 | 387 | 9 | 35 | A1 | 1 | 2 | 0 | 35 | A2 | 1 | 2 | 0 | 100.0% | ML |
| 284 | 458 | 9 | 82 | A1 | 1 | 5 | 0 | 82 | A2 | 1 | 5 | 0 | 94.7% | ML |
| 2974 | 430 | 9 | 82 | A1 | 1 | 5 | 0 | 54 | A2 | 1 | 3 | 0 | 87.2% | ML |
| 3135 | 387 | 10 | 35 | A1 | 1 | 2 | 0 | 35 | A2 | 1 | 2 | 0 | 100.0% | ML |
| 3754 ^d^ | 1200 | 9 | 78 | A1 | 1 | 5 | 0 | 50 | A2 | 1 | 3 | 0 | 95.3% | ML |
| 4370 | 444 | 8 | 82 | A1 | 1 | 5 | 0 | 68 | A2 | 1 | 4 | 0 | 90.2% | ML |
| 44 | 472 | 9 | 96 | A1 | 1 | 6 | 0 | 82 | A2 | 1 | 5 | 0 | 89.3% | ML |
| 4712 | 381 | n.a. | 30 | n.f. | 1 | 2 | 0 | 36 | A2 | 1 | 2 | 0 | 96.7% | ML |
| 523 | 417 | 4 | 54 | A1 | 1 | 3 | 0 | 68 | A2 | 1 | 4 | 0 | 89.4% | ML |
| 527 | 345 | n.a. | 48 | A1 | 1 | 1 | 0 | 38 | n.f. | 0 | 1 | 0 | 94.7% | ML |
| 622 (A) | 414 | 9 | 67 | A1 | 1 | 4 | 0 | 53 | A2 | 1 | 3 | 0 | 85.7% | ML |
| 622 (B) | 370 | n.a. | 39 | A1 | 1 | 2 | 0 | 35 | n.f. | 1 | 2 | 0 | 97.1% | ML |
| 6687 | 444 | 8 | 82 | A1 | 1 | 5 | 0 | 68 | A2 | 1 | 4 | 0 | 88.5% | ML |
| 720 | 378 | 4 | 40 | A1 | 1 | 2 | 0 | 44 | A2 | 1 | 2 | 0 | 93.9% | ML |
| 73 | 415 | 0 | 64 | A1 | 1 | 4 | 0 | 50 | A2 | 1 | 3 | 0 | 86.0% | ML |
| 74 | 416 | 9 | 54 | A1 | 1 | 3 | 0 | 68 | A2 | 1 | 4 | 0 | 89.4% | ML |
| 832 | 415 | 8 | 50 | A1 | 1 | 3 | 0 | 64 | A2 | 1 | 4 | 0 | 95.3% | ML |
| 84 | 387 | 9 | 39 | A1 | 1 | 2 | 0 | 54 | A2 | 1 | 3 | 0 | 90.6% | ML |
| 8712 | 177 | 8 | 39 | A1 | 1 | 2 | 0 | 39 | A2 | 1 | 2 | 0 | 96.9% | ML |
