# Supplementary material for: Tm1: A Mutator/Foldback Transposable Element Family in Root-Knot Nematodes
Source: PLoS One. 2011 Sep 8;6(9):e24534. doi: 10.1371/journal.pone.0024534 (PMC3169594; doi:10.1371/journal.pone.0024534)
Supplement: Table S2 — Tm1 elements of Meloidogyne hapla . TSD length is indicated if both terminal motifs A1 and A2 are present; n.a. (not analyzed) indicates one or both of these motifs is missing; n.f. (not found) indicates that neither motif A1 or A2 was found. GenBank accession numbers, location of Tm1 elements, TSD sequences, and 7 bp terminal motif sequences are provided in Table S4. When possible, elements are oriented with the Left TIR beginning with Motif A1 and the Right TIR terminating with Motif A2. Letters in parenthesis denote Tm1 elements on the same contig. Note: a Identity between TIRs excluding 7 bp terminal motifs. (DOCX) [file pone.0024534.s003.docx]

**Table S2. Tm1 elements of *Meloidogyne hapla***

|  |  |  | **Left TIR** | | | | | **Right TIR** | | | | |  |  |
| --- | --- | --- | --- | --- | --- | --- | --- | --- | --- | --- | --- | --- | --- | --- |
| **Contig** | **Element length (bp)** | **TSD length (bp)** | **Length (bp)** | **Terminal 7 bp** | **B** | **C** | **D** | **Length (bp)** | **Terminal 7 bp** | **B** | **C** | **D** | **Identity between TIRs ^a^** | **Class** |
| 1254 | 1370 | 9 | 151 | A1 | 1 | 9 | 1 | 151 | A2 | 1 | 9 | 1 | 98.60% | Tm1-D |
| 1900 | 1246 | 8 | 131 | A1 | 1 | 7 | 1 | 136 | A2 | 1 | 8 | 1 | 90.50% | Tm1-D |
| 1997 (A) | 1563 | n.a. | 102 | n.f. | 1 | 6 | 1 | 108 | A2 | 1 | 6 | 1 | 100.00% | Tm1-D |
| 1997 (B) | 1581 | 0 | 126 | A1 | 1 | 7 | 1 | 108 | A2 | 1 | 6 | 1 | 91.20% | Tm1-D |
| 2086 | 1170 | 0 | 98 | A1 | 1 | 5 | 1 | 136 | A2 | 1 | 8 | 1 | 88.80% | Tm1-D |
| 2164 | 1009 | 9 | 151 | A1 | 1 | 9 | 1 | 151 | A2 | 1 | 9 | 1 | 100.00% | Tm1-D |
| 2442 | 1534 | 9 | 239 | A1 | 1 | 9 | 1 | 239 | A2 | 1 | 9 | 1 | 99.60% | Tm1-D |
| 659 | 980 | 9 | 151 | A1 | 1 | 9 | 1 | 121 | A2 | 1 | 7 | 1 | 94.60% | Tm1-D |
| 402 | 825 | 9 | 139 | A1 | 1 | 8 | 1 | 95 | A2 | 1 | 5 | 1 | 93.10% | Other |
| 53 | 751 | 9 | 69 | A1 | 1 | 4 | 0 | 97 | A2 | 1 | 6 | 0 | 93.50% | Other |
| 0 | 425 | 9 | 171 | A1 | 1 | 4 | 0 | 154 | A2 | 1 | 3 | 0 | 88.30% | ML |
| 1043 | 430 | 0 | 55 | A1 | 1 | 3 | 0 | 79 | A2 | 1 | 5 | 0 | 93.80% | ML |
| 1076 | 401 | 9 | 51 | A1 | 1 | 3 | 0 | 54 | A2 | 1 | 3 | 0 | 95.50% | ML |
| 123 | 416 | 8 | 51 | A1 | 1 | 3 | 0 | 65 | A2 | 1 | 4 | 0 | 90.90% | ML |
| 1532 | 390 | n.a. | 70 | A2 | 1 | 2 | 0 | 70 | A2 | 1 | 2 | 0 | 91.40% | ML |
| 1597 | 458 | 8 | 79 | A1 | 1 | 5 | 0 | 83 | A2 | 1 | 5 | 0 | 100.00% | ML |
| 1695 | 444 | 9 | 65 | A1 | 1 | 4 | 0 | 83 | A2 | 1 | 5 | 0 | 94.80% | ML |
| 202 | 387 | 10 | 40 | A1 | 1 | 2 | 0 | 40 | A2 | 1 | 2 | 0 | 93.90% | ML |
| 309 | 474 | 8 | 83 | A1 | 1 | 5 | 0 | 97 | A2 | 1 | 6 | 0 | 94.70% | ML |
| 674 | 444 | 9 | 65 | A1 | 1 | 4 | 0 | 83 | A2 | 1 | 5 | 0 | 93.10% | ML |
| 746 | 443 | 9 | 192 | A1 | 1 | 4 | 0 | 198 | A2 | 1 | 5 | 0 | 88.50% | ML |
| 806 | 434 | 8 | 79 | A1 | 1 | 5 | 0 | 55 | A2 | 1 | 3 | 0 | 93.80% | ML |
