# Supplementary material for: Tm1: A Mutator/Foldback Transposable Element Family in Root-Knot Nematodes
Source: PLoS One. 2011 Sep 8;6(9):e24534. doi: 10.1371/journal.pone.0024534 (PMC3169594; doi:10.1371/journal.pone.0024534)
Supplement: Table S3 — Accessory information of Tm1 elements in Meloidogyne incognita . The 7 bp terminal motifs A1 and A2 are shown in boldface. TSDs are underlined. No flanking sequence is shown if TSDs or terminal motifs are not present. Mismatches within the presumed TSDs are shown as lowercase letters. Letters in parenthesis denote Tm1 elements on the same contig. (DOCX) [file pone.0024534.s004.docx]

**Table S3. Accessory information of Tm1 elements in *Meloidogyne incognita***

| **Contig** | **GenBank Accession** | **Location on contig** | **Left TIR motif and TSD** | **Right TIR and TSD** |
| --- | --- | --- | --- | --- |
| 1081 | CABB01001081.1 | 8045 - 7026 | CACA**CGGAAAA** | **CCTACCC**GGGTACAG |
| 119 | CABB01000119.1 | 44860 - 44436 | GGACGGAACA**CGGTTAA** | **CCTACCC**GACGGAACAG |
| 1241 (A) | CABB01001241.1 | 1984 - 3953 | TCTCGGTTA**CGGAAAA** | **CCTACCC**CTCGGTTAA |
| 1241 (B) | CABB01001241.1 | 3956 - 4413 | ...**CGGTTAA** | **CCTACCC**… |
| 1288 | CABB01001288.1 | 9941 - 9565 | not found | not found |
| 1437 | CABB01001437.1 | 11984 - 11545 | TTCCAATTCA**CGGTTAA** | **CCTACCC**TCCAATTCAC |
| 1486 | CABB01001486.1 | 9471 - 9070 | ACTTCGGGGCG**CGGTTAA** | **CCTACCC**CTTCGGGGCGG |
| 163 (A) | CABB01000163.1 | 20725 - 21766 | AGCAGGACTAATG**CGGAAA** | not found |
| 163 (B) | CABB01000163.1 | 22786 - 23201 | CCTCTTGAAA**CGGATAA** | **CCTACCC**CTCTTGAAAT |
| 1763 | CABB01001763.1 | 4317 - 6216 | AATTTTCTTT**CGGTTAA** | **CCTACCC**ATTTTCTTTA |
| 1830 | CABB01001830.1 | 9063 - 8607 | ATAAGCTTTT**CGGTTAA** | **CCTACCC**TAAGCTTTTT |
| 1831 | CABB01001831.1 | 2690 - 3556 | AGAAAAAATA**CGGTAAA** | **CCTACCC**GAAAAAATAA |
| 206 | CABB01000206.1 | 30171 - 29728 | GTAAAATAGG**CGGTTAA** | **CCTACCC**TAAAATAGGT |
| 2092 | CABB01002092.1 | 3837 - 4729 | GTATTTATCT**CGGAAAA** | **CCTACCC**TATTTATCTA |
| 2158 | CABB01002158.1 | 2804 - 2403 | TGCTAGAAT**CGGTTAA** | **CCTACCC**GCTAGAATA |
| 2269 | CABB01002269.1 | 4698 - 4296 | CAATTCTCCG**CGGTAAA** | **CCTACCC**AATTCTCCGG |
| 2332 | CABB01002332.1 | 2103 - 1725 | TCATCAAATT**CGGTTAT** | **CCTACCC**CATCAAATTC |
| 273 | CABB01000273.1 | 26668 - 26282 | GCAGAAAAAC**CGGTTAA** | **CCTACCC**CCAGAAAAACG |
| 274 | CABB01000274.1 | 33966 - 31374 | AAACTAACTA**CGGTTAA** | **CCTACCC**AACTAACTAC |
| 284 | CABB01000284.1 | 31883 - 31426 | TTAATACTGT**CGGTTAA** | **CCTACCC**TAATACTGTA |
| 2974 | CABB01002974.1 | 3914 - 4343 | AAATATATTA**CGGTTAA** | **CCTACCC**AATATATTAG |
| 3135 | CABB01003135.1 | 7720 - 7334 | ACCgGAACTCC**CGGTTAA** | **CCTACCC**CCcGAACTCCA |
| 3754 | CABB01003754.1 | 2707 - 1508 | AAAAAAAGTC**CGGTTAA** | **CCTACCC**AAAAAAGTCT |
| 4370 | CABB01004370.1 | 4050 - 4493 | CTGACAAAA**CGGTTAA** | **CCTACCC**TGACAAAAT |
| 44 | CABB01000044.1 | 43208 - 42737 | GTGGATTGGA**CGGTTAA** | **CCTACCC**TGGATTGGAA |
| 4712 | CABB01004712.1 | 2703 - 3083 | not found | **CTTCCCC**… |
| 523 | CABB01000523.1 | 18650 - 18234 | ACGCTCA**CGGTTAA** | **CCTACCC**CGCTT |
| 527 | CABB01000527.1 | 21296 - 21640 | ...**CGGATAA** | not found |
| 6020 | CABB01006020.1 | 960 - 1842 | not found | **CCTACCC**GCTGTCATGTTTG |
| 622 (A) | CABB01000622.1 | 1507 - 1920 | ATCAAAAAGT**CGGTTAA** | **CCTACCC**TCAAAAAGTT |
| 622 (B) | CABB01000622.1 | 19104 - 19469 | not found | not found |
| 6687 | CABB01006687.1 | 1532 - 1089 | CTAAATTTG**CGGTTAA** | **CCTACCC**TAAATTTGC |
| 720 | CABB01000720.1 | 2608 - 2231 | TAAACCTA**CGGTTAA** | **CCTAAAC**CAAAACG |
| 73 | CABB01000073.1 | 13190 - 13604 | AATT**CGGATGA** | **CCTACCC**GTAATGACATATT |
| 74 | CABB01000074.1 | 4101 - 4516 | TATTTATTTG**CGGTTAA** | **CCTACCC**ATTTATTTGG |
| 832 | CABB01000832.1 | 11370 - 10956 | AGGCGCAAA**CGGTTAA** | **CCTACCC**GGCGCAAAG |
| 84 | CABB01000084.1 | 53569 - 53183 | CCTGGAAATG**CGGTTAA** | **CCTACCC**CTGGAAATGA |
| 847 | CABB01000847.1 | 16292 - 15416 | ACTCTAACTT**CGGTAAA** | **CCTACCC**CTCTAACTTG |
| 8712 | CABB01008712.1 | 12 - 188 | TTCTTTTTG**CGGTTAA** | **CCTACCC**TCTTTTTGT |
